# Occurrence and Clinical Relevance of *Mycobacterium chimaera* sp. nov., Germany

**DOI:** 10.3201/eid1409.071032

**Published:** 2008-09

**Authors:** Birgitta Schweickert, Oliver Goldenberg, Elvira Richter, Ulf B. Göbel, Annette Petrich, Petra Buchholz, Annette Moter

**Affiliations:** Charité-Universitätsmedizin, Berlin, Germany (B. Schweickert, A. Petrich, U.B. Göbel, P. Buchholz, A. Moter); Transgenomic Ltd., Glasgow, Scotland, UK (O. Goldenberg ); Nationales Referenzzentrum für Mykobakterien am Forschungszentrum, Borstel, Germany (E. Richter)

**Keywords:** Mycobacterium avium complex, Mycobacterium avium-intracellulare infection, atypical mycobacterium infections, high-performance liquid chromatography, ribosomal DNA, ribosomal spacer DNA, molecular diagnostic techniques, molecular epidemiology, dispatch

## Abstract

Retrospective molecular genetic analysis of 166 *Mycobacterium intracellulare* isolates showed that 143 (86%) strains could be assigned to *Mycobacterium chimaera* sp. nov. Of 97 patients from whom *M. chimaera* sp. nov. was isolated, only 3.3% exhibited mycobacterial lung disease, whereas all *M. intracellulare* isolates caused severe pulmonary infections.

Bacteria of the *Mycobacterium avium* complex (MAC) play an important role among infections caused by nontuberculous mycobacteria (NTM). MAC consists of the 2 well-established species, *M. avium* (which has 4 subspecies) and *M*. *intracellulare*, as well as several other closely related mycobacteria (*1*). Recently, a new species derived from the group of unnamed members of the MAC has been defined. It combines features characteristic of different MAC members and has been named *M. chimaera* sp. nov. (*2*).

Based on the sequence of the 16–23S internal transcribed spacer (ITS) region, this species genetically corresponds to sequevar MAC-A and differs from *M. intracellulare* type strain, sequevar MIN-A (DSMZ 43223) by 20 nt mismatches (*2**,**3*). In contrast, the 16S rRNA gene sequence is identical, except for 1 nt mismatch, with that of the *M. intracellulare* type strain. Because sequencing of the 16S rDNA still is considered the approved standard for the identification of NTMs, *M. chimaera* sp. nov. usually has been misreported as *M. intracellulare*. Molecular genetic standard tools in clinical microbiologic laboratories do not differentiate MAC members. These tools merely provide a rough classification in *M. intracellulare* and *M. avium* and/or the MAC group as a whole. Currently, a detailed genotyping of MAC is restricted to research laboratories. Nevertheless, several studies have shown that certain serotypes or genotypes were associated with different clinical manifestations of MAC infection concerning the patient groups affected, the localization and course of disease, and the antimicrobial drug resistance patterns (*4**,**5*).

## The Study

Since available data on the epidemiology of *M. chimaera* sp. nov. are sparse, we performed a retrospective study to determine the frequency of its occurrence within the group of MAC-positive clinical specimens and its possible role in causing human disease in comparison to *M. intracellulare*. We reanalyzed mycobacterial isolates from 97 in-house patients of the Charité University Hospital that have been processed in our laboratory from 2002 through 2006. An additional 69 isolates were provided by the National Reference Center (NRC) for Mycobacteria in Borstel, Germany. All strains had previously been classified as *M. intracellulare* by 16S rDNA–based methods. In addition to the partial 16S rRNA gene, we sequenced the 16S–23S ITS region to allow for unambiguous identification. Amplification of the partial 16S rRNA gene was performed according to a standard procedure (*6*). For the amplification of the ITS, the following primers were used: Sp1 (5′-ACC TCC TTT CTA AGG AGC ACC-3′) and Mb23S.44n (5′-TCT CGA TGC CAA GGC ATC CAC C-3′) (*7**,**8*). PCR conditions and the sequencing procedure are described elsewere (*9*). The assignment to sequevars was based on the ITS sequence, according to the taxonomy introduced by Frothingham and Wilson (*3*). Laboratory analysis was performed without knowledge of the clinical course of the disease. The frequency distribution of the 166 strains according to their species or sequevar designations is presented in Table 1.

**Table 1:** Distribution of mycobacterial species and sequevars*

Species, sequevar†	Total isolates, no. (%), n = 166	Isolates from Charité,‡ no. (%), n = 97	Isolates from NRC,‡ no. (%), n = 69	Odds ratio (p value§)
MAC				
MAC-A	143 (86.1)	90 (92.8)	53 (76.8)	3.88 (p = 0.003)
MAC-C	2 (1.2)	1 (1.0)	1 (1.5)	ND
MAC-E	1 (0.6)	1 (1.0)	0	ND
Min				
Min-A	17 (10.2)	3 (3.1)	14 (20.3)	0.12 (p<0.001)
Min-C	3 (1.8)	2 (2.1)	1 (1.5)	ND

*MAC, *M. avium* complex species; MAC-A, *M. chimaera* sp. nov.; ND, not done; Min, *M. intracellulare*; Min-A, *M. intracellulare* type strain. †Classification of MAC strains according to the taxonomy of Frothingham and Wilson (*3*). ‡Charité, Charité University Hospital, Berlin, Germany; NRC, National Reference Center for Mycobacteria, Borstel, Germany. §χ^2^ test, software package Stata version 9 (Stata Corporation, College Station, TX, USA).

In addition, we tested the application of denaturating high-performance liquid chromatography (DHPLC) for the identification of the ITS PCR product to distinguish *M. intracellulare,* type strain, and *M. chimaera* sp. nov. DHPLC is a semiautomated, quick, and sensitive technique and has been used for the detection of genetic variations predominantly for genotyping purposes of a wide range of human diseases (*10*). Recently, it has also been introduced for the identification and genotyping of bacterial species (*11*) and yeasts (*12*). The amplified ITS gene fragments were separated on the WAVE 3500 HT System (Transgenomic, Omaha, NE, USA). Optimal separation was achieved at an oven temperature of 61.5°C and a flow rate of 1.4 mL/min on an integrated DNASep HT cartridge. Samples were loaded in 53.5% buffer A (0.1 mmol/L triethylammoniumacetate [TEAA]) and 46.5% buffer B (0.1 mmol/L TEAA in 25% acetonitrile). After 30 s, buffer B was set to 51.5%, reaching 60.5% after an additional 4.5 min. The column was cleaned with 100% buffer B (5.0–5.6 min) and equilibrated with 46.5% buffer B (5.7–6.6 min) before the next injection. Analysis was accomplished with Navigator software version 1.5.4 (Transgenomic).

Both reference strains, *M. intracellulare,* sequevar Min-A (DSMZ 43223), and *M. chimaera* sp. nov., sequevar MAC-A (DSMZ 44623), showed reproducible peak profiles. *M. avium* spp. *avium*, sequevar Mav-A, DSMZ 44156 (21 mismatches to *M. intracellulare*, sequevar Min-A; 18 mismatches to *M. chimaera* sp. nov.,) and *M. intracellulare*, sequevar Min-C (2 mismatches to *M. intracellulare,* sequevar Min-A; 14 mismatches to *M. chimaera* sp. nov.) served as negative run controls providing a different peak location. All clinical *M. intracellulare*, sequevar Min-A strains, and *M. chimaera* sp. nov. strains (sequevar MAC-A) could be allocated unequivocally to the 2 reference strains by their congruent peak patterns (Figure). *M. avium* complex isolates, sequevars MAC-C and MAC-E, and *M. intracellulare* isolates, sequevar Min-C, showed different peak profiles that could easily be separated from the 2 reference species. All DHPLC results could be reproduced in a second run conducted on another day.

**Figure:**
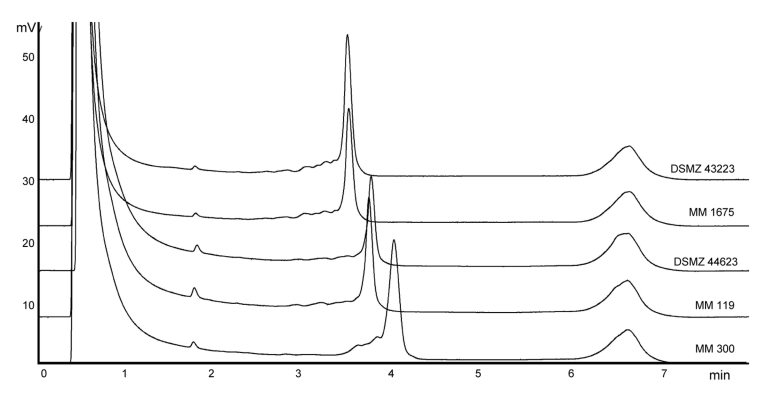
Denaturating high-performance liquid chromatography profiles after separation of PCR-amplified internal transcribed spacer regions of *Mycobacterium* spp. Strain designations from above: DSMZ 43223, *M. intracellulare*, sequevar MIN-A type strain; MM 1675, *M. intracellulare*, sequevar MIN-A, patient strain; DSMZ 44623, *M. chimaera* sp. nov., sequevar MAC-A type strain; MM 119; *M. chimaera* sp. nov., sequevar MAC-A, patient strain; MM 300, *M. intracellulare*, sequevar MIN-C, patient strain.

The clinical relevance of *M. intracellulare/chimaera* sp. nov. strains isolated from respiratory specimens of 97 in-house patients of the Charité University Hospital has been assessed according to the 1997 American Thoracic Society criteria for NTB lung disease (*13*). The data have been drawn from past hospital records. Cases were subdivided into 3 categories: clinically relevant, clinically not relevant, and undetermined (Table 2). A clinical follow up of the 69 isolates provided by the NRC for mycobacteria was not possible. The characteristics of the patients with mycobacterial infection resemble already known features, such as underlying lung disease, immunosuppression, female sex, and microscopically positive respiratory samples (Appendix Table).

**Table 2:** Distribution of MAC isolates according to ATS criteria*

Mycobacterial species, sequevars†	Total no. (%)	Clinically relevant,‡ no. (%)	Clinically not relevant,§ no. (%)	Clinical relevance undetermined,¶ no. (%)
MAC				
MAC-A	90 (100)	3 (3.3)	82 (91.1)	5 (5.6)
MAC-C	1 (100)	0	1 (100)	0
MAC-E	1 (100)	0	1 (100)	0
Min				
Min-A	3 (100)	3 (100)	0	0
Min-C	2 (100)	0	2 (100)	0
Total	97 (100)	6 (6.2)	86 (88.7)	5 (5.2)

*ATS, American Thoracic Society; MAC, *Mycobacterium avium* complex; MAC-A, *M. chimaera* sp. nov.; Min, *M. intracellulare*. Min-A, *M. intracellulare* type strain. †Classification of MAC strains according to the taxonomy of Frothingham and Wilson (*3*). ‡ATS criteria (*13*) for mycobacterial lung disease are fulfilled. §ATS criteria for mycobacterial lung disease are not fulfilled. ¶ATS criteria for mycobacterial lung disease are fulfilled, but radiologic findings have been attributed to the underlying illness or insufficient sample numbers.

## Conclusions

DHPLC was a rapid and reliable method for distinguishing *M. intracellulare* type strain from *M. chimaera* sp. nov. within a well-defined group of mycobacterial isolates. Low costs and the high degree of automation predispose this technique for epidemiologic studies. Our results show that *M. chimaera* sp. nov. accounts for most of the mycobacterial isolates formerly classified as *M. intracellulare*. The small number of clinically relevant isolates (3.3%) suggests relatively low pathogenicity. As most other studies assessing the pathogenic potential of clinical NTM isolates referred either to members of the whole MAC group (inclusively *M. avium*) or to the complete NTM spectrum, their results cannot be compared (*14*). Our observations are not concordant with those of Tortoli et al. (who suspected that *M. chimaera* sp. nov. was highly virulent), possibly because of the low number of cases analyzed (12 patients) (*2*). The most striking result of our study was that all 3 *M. intracellulare,* sequevar Min-A, isolates were unequivocally associated with severe mycobacterial lung disease. Despite the low case numbers, these findings suggest that this species is more virulent and justify further epidemiologic investigations to verify this observation.

In agreement with other authors, our observations indicate that a precise differentiation of MAC isolates may provide clinically relevant data (*4**,**5*). This conclusion is in accord with a recently published review that discusses advances and future aspects of MAC genomics and points out the importance of taking into account the heterogeneity of MAC species (*15*).

If one assumes substantial differences in pathogenicity, the allocation of MAC isolates to defined species may facilitate diagnosis of mycobacterial lung disease. However, implementation of a staged identification procedure in routine microbiologic laboratories requires the availability of commercial, easy-to-use test kits. Because diagnosis of NTM infections remains a challenge and often results in indecisive situations that prolong the administration of adequate therapy, the rapid identification of MAC-related species highly predictive for mycobacterial disease would be very useful.

## Supplementary Material

Appendix Table Comparison of characteristics of Mycobacterium chimaera sp. nov. and M. intracellulare, sequevar Min-A-positive patients
