# Supplementary material for: Occurrence and Clinical Relevance of Mycobacterium chimaera sp. nov., Germany
Source: Emerg Infect Dis. 2008 Sep;14(9):1443–6. doi: 10.3201/eid1409.071032 (PMC2603105; doi:10.3201/eid1409.071032)
Supplement: Appendix Table — Comparison of characteristics of Mycobacterium chimaera sp. nov. and M. intracellulare, sequevar Min-A-positive patients [file 07-1032_appT-s1.pdf]

Appendix Table. Comparison of characteristics of *Mycobacterium chimaera* sp. nov. and *M. intracellulare,* sequevar Min-A–positive patients

	Clinical relevance			
	M. chimaera sp. nov.			*M. intracellulare**
Characteristic	Not relevant, no (%), n = 82	Intermediate, no (%), n = 5	Relevant, no. (%), n = 3	Relevant, no (%), n = 3
Age, y				
<60	38 (46.3)	2 (40)	2 (66.7)	1 (33.3)
≥60	44 (53.7)	3 (60)	1 (33.3)	2 (66.7)
Sex				
F	20 (24.4)	2 (40)	2 (66.7)	3 (100)
M	62 (75.6)	3 (60)	1 (33.3)	0
Clinical symptoms				
Respiratory and general†	71 (86.6)	5 (100)	3(100)	3 (100)
General alone	11 (13.4)	0	0	0
Radiologic findings‡	63 (76.8)	5 (100)	3(100)	3 (100)
Underlying disease§				
Lung¶	34 (41.5)	3 (60)	3 (100)	3 (100)
Lung cancer	12 (14.6)	0	0	0
Non–lung cancer	9 (11.0)	4 (80)	0	0
Autoimmune disease#	3 (3.7)	0	2 (66.7)	0
Other	18 (22.0)	0	0	0
Not known	6 (7.3)	0	0	0
Immunosuppression				
HIV	7 (8.5)	1 (20)	0	0
Immunosuppressive therapy	6 (7.3)	2 (40)	3 (100)	0
Hemato-oncologic disease	4 (4.9)	3 (60)	0	0
Other pathogens				
Active tuberculosis	5 (6.1)	0	0	0
Former tuberculosis	3 (3.7)	0	0	0
M. gordonae	6 (7.3)	0	0	0
Bacterial, viral, fungal**	15 (18.3)	0	0	0
Smear positive	0	0	2 (66.7)	2 (66.7)
PCR positive††	0	0	3 (100)	3 (100)
Single isolate	68 (82.9)	3 (60)	0	0
Multiple isolates	14 (17.1)	2 (40)	3 (100)	3 (100)

*All *M. intracellulare*, sequevar Min-A, isolates were clinically relevant.
†Respiratory symptoms: cough, expectoration, dyspnea, hemoptysis; general symptoms: fever, fatigue, weight loss, night sweats.
‡Infiltrations, bronchiectasis, fibrosis, fibronodular lesions, cavitation, unclear focal lesion, tumor.
§Multiple answers possible.
¶Chronic obstructive pulmonary disease, asthma bronchiale, pulmonary fibrosis, bronchiectasis.
#Sclerodermia, rheumatoid arthritis, ankylosing spondylitis (Morbus Bechterew).
***Streptococcus pneumoniae, Streptococcus pyogenes, Staphylococcus aureus, Bordetella pertussis, Serratia marcescens, Salmonella enteritidis, Legionella pneumophila, Cytomegalovirus, Aspergillus fumigatus, Cryptococcus neoformans.*
††PCR directly from the clinical sample has been performed for 30 patients.
